# Effect of Polysaccharide Sources on the Physicochemical Properties of Bromelain–Chitosan Nanoparticles

**DOI:** 10.3390/polym11101681

**Published:** 2019-10-15

**Authors:** Janaína Artem Ataide, Eloah Favero Gérios, Letícia Caramori Cefali, Ana Rita Fernandes, Maria do Céu Teixeira, Nuno R. Ferreira, Elias Basile Tambourgi, Angela Faustino Jozala, Marco Vinicius Chaud, Laura Oliveira-Nascimento, Priscila Gava Mazzola, Eliana B. Souto

**Affiliations:** 1 Graduate Program in Medical Sciences, School of Medical Sciences, University of Campinas (UNICAMP), 13010 Campinas, Brazil; janaina.a.ataide@gmail.com; 2 Department of Pharmaceutical Technology, Faculty of Pharmacy, University of Coimbra (FFUC), Pólo das Ciências da Saúde, 3000 Coimbra, Portugal; eloahfaverogerios@gmail.com (E.F.G.); anaritavfernandes@gmail.com (A.R.F.); mceuteixeira1@gmail.com (M.d.C.T.); ricardao@ci.uc.pt (N.R.F.); 3 Faculty of Pharmaceutical Sciences, University of Campinas (UNICAMP), 13010 Campinas, Brazil; laura.nascimento@fcf.unicamp.br; 4 Graduate Program in Biosciences and Technology of Bioactive Products, Institute of Biology, University of Campinas (UNICAMP), 13010 Campinas, Brazil; 5 School of Chemical Engineering, University of Campinas (Unicamp), 13010 Campinas, Brazil; eliastam@feq.unicamp.br; 6 Laboratory of Industrial Microbiology and Fermentation Process, University of Sorocaba (UNISO), 18010 Sorocaba, Brazil; angela.jozala@prof.uniso.br; 7 Laboratory of Biomaterials & Nanotechnology, University of Sorocaba (UNISO), 18010 Sorocaba, Brazil; marco.chaud@prof.uniso.br; 8 CEB - Centre of Biological Engineering, University of Minho, Campus de Gualtar, 4700 Braga, Portugal

**Keywords:** bromelain, chitosan nanoparticles, physicochemical characterization, low molecular weight chitosan, chitosan oligosaccharide lactate, chitosan from shrimp shells.

## Abstract

Bromelain, a set of proteolytic enzymes potential pharmaceutical applications, was encapsulated in chitosan nanoparticles to enhance enzyme stability, and the effect of different chitosan sources was evaluated. Chitosan types (i.e., low molecular weight chitosan, chitosan oligosaccharide lactate, and chitosan from shrimp shells) produced nanoparticles with different physicochemical properties, however in all cases, particle size and zeta potential decreased, and polydispersity index increased after bromelain addition. Bromelain encapsulation was higher than 84% and 79% for protein content and enzymatic activity, respectively, with low molecular weight chitosan presenting the highest encapsulation efficiency. Nanoparticle suspension was also tested for accelerated stability and rheological behavior. For the chitosan–bromelain nanoparticles, an instability index below 0.3 was recorded and, in general, the loading of bromelain in chitosan nanoparticles decreased the cohesiveness of the final suspension.

## 1. Introduction

Nanoparticulated systems have been widely used for a range of applications in drug delivery [1], as they offer modified release, localized release, and protection of the loaded active ingredient against degradation [2]. Polysaccharide nanoparticles gained special attention as drug delivery systems due to their biocompatibility and biodegradability, and availability of different production methods [2,3]. Among the different types of polysaccharides used in the production of nanoparticles, chitosan is of particular interest because of its availability in nature, biocompatibility, biodegradability, mucoadhesive properties, easy surface modification, and low toxicity [2,3]. In addition, chitosan exhibits antibacterial, antifungal and antitumor activities, haemostatic properties, accelerates wound healing [4,5], and stimulates the immune system [1,6,7,8].

Chitosan is obtained from the deacetylation of chitin, which is derived from crustacean shells and fungi cell walls, being one of the most abundant natural polysaccharides [2,9,10,11]. Reaction conditions, extent, and chitin source are factors that can be modified during the chitin deacetylation process. These modifications change the characteristics of the obtained polysaccharide, such as molecular weight, pKa, and deacetylation degree, changing chitosan’s physical and chemical properties [2,12]. Chitosan nanoparticles have been used as carrier for genes, proteins, vaccines, and antiallergic, antiviral, and anticancer drugs, through various administration routes, e.g., oral, intravenous, nasal, vaginal, and ocular [9,12,13,14,15,16]. Proteins are easily degraded in vivo by enzymes, having poor stability and a short half-life, as well as poor permeability. Chitosan nanoparticles have been proposed as protein carriers, promoting the contact between protein and cell membrane and modifying the protein release profile [12,17,18].

Bromelain is a set of proteolytic enzymes found in Bromeliaceae family, mainly in pineapple (*Ananas comosus* L.), with potential pharmaceutical properties such as anti-inflammatory, antithrombotic, fibrinolytic, antitumor activity, and immunomodulatory effect [19,20,21]. Bromelain has been investigated in wound healing and as debridement agent [22,23,24]. Previous studies showed bromelain instability when directly applied as topical formulations, even when stored at low temperatures [25,26,27], pointing out that bromelain would benefit from chitosan-based encapsulation. 

A recent review discusses the uses of bromelain and its loading into nanoparticles obtained from different materials, including synthetic and natural polymers [28]. However, bromelain has not been used only as encapsulated active, playing different roles in pharmaceutical nanotechnology field, including when mixed with chitosan. Bromelain has been used as surface modifier in lactobionic acid-modified chitosan nanoparticles to improve their tumor penetration ability [29,30]. Bromelain has also been encapsulated using linoleic acid modified carboxylmethyl chitosan, improving its thermal stability and maintaining its catalytic activity [31].

In this work, we propose the loading of bromelain in chitosan nanoparticles obtained from different sources, as an innovative approach for the development of a wound healing formulation. The effect of the chitosan source (low molecular weight chitosan (LMW), chitosan from shrimp shells (SHR) and chitosan oligosaccharide lactate (LAC)) on the physicochemical parameters of bromelain-loaded nanoparticles has also been studied. 

## 2. Materials and Methods

### 2.1. Materials

Bromelain from the pineapple stem, azocasein, Bradford reagent, low molecular weight chitosan (molecular weight 50–190 kDa, 75–85% deacelylated), chitosan from shrimp shells (molecular weight 150 kDa, 95% deacetylated, low viscosity <200 mPa·s), and chitosan oligosaccharide lactate (molecular weight 4–6 kDa, >90% deacetylated, 60% of oligosaccharide) were purchased from Sigma-Aldrich^®^ (St. Louis, MO, USA). All the other purchased reagents were of analytical grade. 

### 2.2. Standard Solution of Bromelain

Bromelain standard solution (10 mg/mL) was prepared by dissolving bromelain in distilled water and filtered using 0.22 µm Millipore™ membrane (Sigma-Aldrich, Sintra, Portugal).

### 2.3. Protein Concentration and Enzymatic Activity

Total protein concentration was determined following the method described by Bradford [32]. For bromelain enzymatic activity measurement, azocasein was used as substrate [33,34] at 37 °C for 10 min. The reaction was then interrupted by the addition of trichloroacetic acid. The obtained mixture was centrifuged and absorbance of a supernatant aliquot (200 µL) was measured at 440 nm in a microplate reader (Synergy™ HT, BioTek Instruments Inc., Winooski, VT, USA). Enzymatic activity was then calculated in activity units (U/mL), which is the amount that caused an increase of one unit in absorbance of 1 mL of sample in 60 min.

### 2.4. Nanoparticles Production with Different Chitosan Types

Nanoparticles were produced by an ionic crosslinking technique [10], using sodium tripolyphosphate (TPP) as the crosslinking agent, in 30% (*w*/*w*) concentration ratio of TPP to total chitosan amount, and mechanical stirring at approximately 350 rpm (Multistirrer 15 Magnetic Stirrer, Velp Scientific Inc., Bohemia, NY, USA). In general, for each 4 mL of chitosan solution at 2.5 mg/mL (in acetic acid 1%, *v*/*v*, pH 5.0, previously filtered through 0.45 μm Millipore™ membranes), 6 mL of TPP solution at 0.5 mg/mL (filtered through 0.22 μm) was added dropwise (30 s). Immediately after the addition of TPP, 1 mL of standard bromelain solution was added to produce chitosan–bromelain nanoparticles, or 1 mL of distilled water to produce bromelain-free chitosan nanoparticles (blank nanoparticles). After the addition of all components, the solution was kept under magnetic stirring for 40 min. To compare different specifications and origin, three chitosan types were used for nanoparticles production, i.e., low molecular weight chitosan (LMW), chitosan from shrimp shells (SHR), and chitosan oligosaccharide lactate (LAC).

### 2.5. Nanoparticles Characterization

#### 2.5.1. Dynamic Light Scattering (DLS) and Zeta Potential

The mean particle size (Z-ave), polydispersity index (PdI), and zeta potential (ZP) of produced nanoparticles were determined using the Zetasizer Nano ZS equipment (Malvern Panalytical Ltd., Royston, UK). The Z-ave and PdI were determined by dynamic light scattering (DLS) at 25 °C, and the results are expressed as the average and standard deviation of 10 consecutive runs. The ZP was determined using laser Doppler microelectrophoresis, at 25 °C and the results are expressed as the average and standard deviation of 3 measurements. The DLS conditions are displayed in Table 1.

#### 2.5.2. Nanoparticles Tracking Analysis (NTA)

Nanoparticles tracking analysis (NTA) was also used to determine the nanoparticle’s mean diameter and size distribution, using NanoSight NS300 (Malvern Panalytical Ltd., Royston, UK) equipment. This technique enables the determination of nanoparticles concentration expressed as number of particles/mL. Prior to analysis, nanoparticle suspensions were diluted 1000 times in Milli-Q water.

#### 2.5.3. Encapsulation Parameters and Enzymatic Activity

To determine the bromelain loading capacity (LC%) and encapsulation efficiency (EE%), nanoparticles were centrifuged (Centrifuge 5810R, Eppendorf, Hamburg, Germany) for 10 min at 14,000 g in 0.5 mL ultrafiltration tubes with a 100 kDa membrane (Amicon^®^ Ultra 100k, Millipore, Merck KGaA, Darmstadt, Germany). The quantification of the protein was determined as described in Section 2.3, in the initial bromelain solution, in the filtrate, and in the nanoparticle suspensions. Nanoparticles without bromelain were submitted to the same process and used as a blank control. The LC% was calculated as the ratio between the mass of bromelain loaded into nanoparticles and the mass of chitosan used for their production, applying Equation (1). The EE% was determined as the difference in protein concentration in the initial bromelain solution and in the filtered solution using Equation (2). The Enzymatic Activity (EA%) was determined as the difference in enzymatic activity measured in the initial bromelain solution and in the filtrate using Equation (3).

(1) $$LC\%=\frac{Mass\;of\;bromelain\;loaded\;into\;nanoparticles}{Mass\;of\;chitosan\;used\;for\;production\;of\;nanoparticles}\times 100$$

(2) $$EE\%=\frac{\left[Bromelain\;in\;initial\;solution\right]-\left[Bromelain\;in\;filtrate\right]}{\left[Bromelain\;in\;initial\;solution\right]}\times 100$$

(3) $$EA\%=\frac{Activity\;of\;bromelain\;in\;initial\;solution-Activity\;of\;bromelain\;in\;filtrate}{Activity\;of\;bromelain\;in\;initial\;solution}\times 100$$

#### 2.5.4. Scanning Electron Microscopy (SEM)

The morphological characteristics of the nanoparticles were observed using a scanning electron microscope LEO 440i with X-ray dispersive energy detector 6070 (LEO Electron Microscopy, Cambridge, UK). Images were obtained using an acceleration voltage of 15 kV. Prior to microscopic analysis, samples were diluted in water (1:1000, *v*/*v*) dripped on stub and dried at room temperature under vacuum. Dried samples were coated with gold (92 A°) using SC7620 Sputter Coater Polaron (VG Microtech, Kent, UK).

#### 2.5.5. Fourier Transform Infrared (FTIR)

Infrared spectra of nanoparticles with and without bromelain were obtained in an infrared spectrophotometer with Fourier transform (Shimadzu Scientific Instruments, Model 8300, Kyoto, Japan), operating at 4000 to 650 cm^−1^, with 4 cm^−1^ resolution. Prior to infrared analysis, nanoparticles were freeze-dried overnight. Bromelain, TPP, and chitosans were also characterized by FTIR technique for data comparison.

### 2.6. Nanoparticles Long-Term Stability

Nanoparticles long-term stability (up to 30 days) was extrapolated from short-term measurements (i.e., of 33 min) performed by a dispersion analyzer of multiwavelength LUMiSizer^®^ (LUM GmbH, Berlin, Germany). LUMiSizer applies a Relative Centrifugal Force (RCF) from 5 to 2325 RCF, which accelerates the movement of materials in relation to gravity, and records the instability index using the SEPView^®^ software (LUm GmbH, Version 6, Berlin, Germany). Samples of chitosan and chitosan–bromelain nanoparticles were submitted to this analysis [35].

### 2.7. Texture Analysis

Mechanical properties of nanoparticle suspensions were evaluated using a TA.XT Plus Texture Analyser (Stable Micro Systems Ltd.a., Godalming, UK) with a Back Extrusion Rig platform. This platform comprises a sample container, which is centrally located below a disk plunger responsible for performing a compression test leading to the extrusion of the product. Firmness (g), consistency (gs), cohesiveness (g), and viscosity index (gs) parameters were recorded using the instrument software based on graph of force (g) as a function of time (s).

### 2.8. Statistical Analysis

All measurements were performed in triplicate, and all results are expressed as mean ± standard deviation values. Statistical significance was established at *p* < 0.05, and was calculated using one-way analysis of variance or Student′s T-test.

## 3. Results and Discussion

### Production and Characterization of Nanoparticles

**Figure 1:**
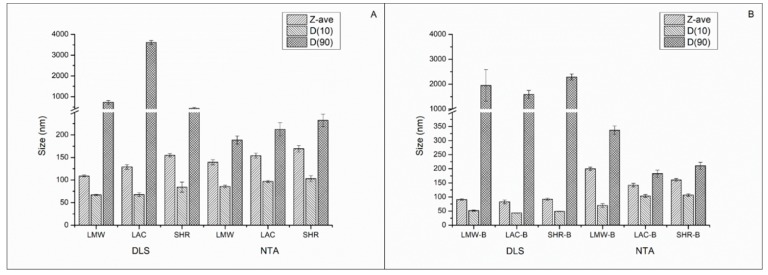
Chitosan (**A**) and chitosan-bromelain (**B**) nanoparticles mean size obtained by DLS and nanoparticle tracking analysis (NTA). LMW = low molecular weight chitosan nanoparticles; LAC = chitosan oligosaccharide lactate nanoparticles; SHR = chitosan from shrimp shells nanoparticles; B = bromelain; Z-ave = mean diameter; D(10) = size below which 10% of material is contained; D(90) = size up to and including which 90% of material is contained.

The selection of the chitosans for the production of the nanoparticles has been based on the differences in molecular weight, degree of acetylation, and substituents. Nanoparticles with and without bromelain produced with different chitosan types were physically characterized by DLS, laser Doppler microelectrophoresis, and by NTA (Figure 1 and Table 2).

The obtained results show that, regardless the type of chitosan used, the addition of bromelain leads to a decrease of the Z-ave and ZP of nanoparticles, when compared to the respective blank nanoparticles. According to Hebbar et al. [36], at pH 5.0 bromelain is negatively charged, which may favor the electrostatic interaction with chitosan positively charged amine groups, decreasing both the size and the surface charge of nanoparticles. Except for LAC nanoparticles, the loading of bromelain led to the increase of the PdI. Theoretically, the PdI for monodispersed nanoparticles should be zero, however a PdI lower than 0.1 can also be considered monodispersed, while systems with a PdI from 0.1 to 0.4 are considered moderately polydispersed [37]. When measuring the D90 of bromelain-loaded nanoparticles prepared with LMW and SHR chitosans, this parameter decreased when recorded by NTA and increased when recorded by DLS, in comparison to the blank nanoparticles obtained with the same type of chitosans. It is worth noticing that in DLS the contribution of each particle to size result relates to its volume, while in NTA it is the number of particles that contributes to the percentage that each size class occupies of the overall distribution. Volume distribution is therefore more realistic and is particularly useful when the presence and distribution of large particles have to be monitored. In the present work, we see that only LMW-bromelain nanoparticles can be accepted and considered moderate polydispersed systems, while the other chitosan–bromelain nanoparticles are highly polydispersed systems.

To determine the loading capacity and encapsulation efficiency, nanoparticles were centrifuged in ultrafiltration tubes and the quantification of protein and enzymatic activity determined in the filtered solutions. The LC%, EE%, and EA% were calculated using Equations (1), (2), and (3), respectively, and results are depicted in Table 3. For all nanoparticles, the EE% was recorded above 84% and the EA% above 79%. LAC-B nanoparticles showed the highest EE% (97.7%), while LWM-B nanoparticles showed the highest enzymatic activity (91.9%). Considering the hydrophilic character of bromelain, these results are promising and confirm the loading of the protein in chitosan nanoparticles, as also demonstrated by FTIR analysis (see below). In addition, the electrostatic interaction of bromelain with the positively charged amine groups of chitosan is favored because at pH 5.0 the protein is negatively charged. 

**Figure 2:**
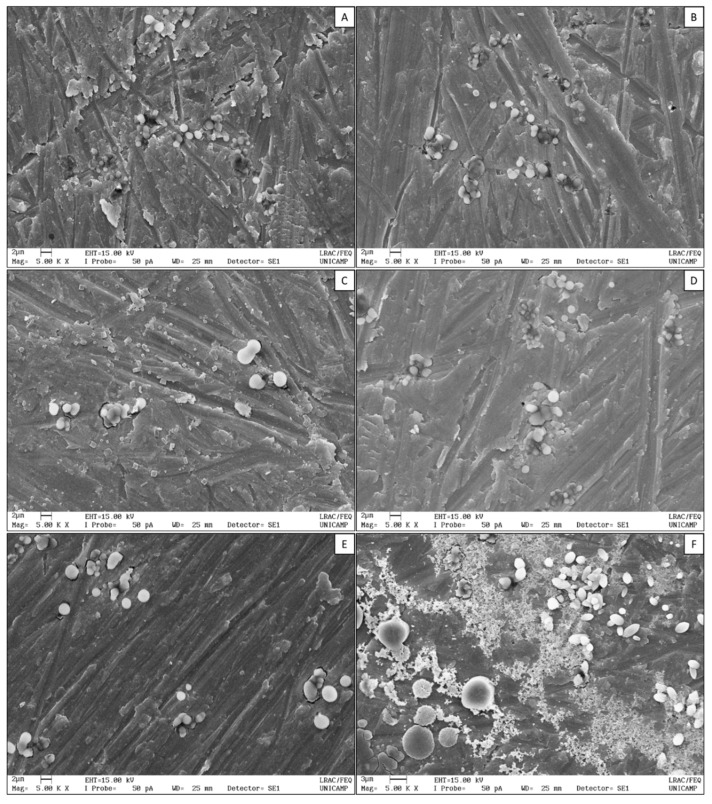
Scanning electron microscopy (SEM) images of LMW (**A**), LMW-B (**B**), LAC (**C**), LAC-B (**D**), SHR (**E**), and SHR-B (**F**) nanoparticles. LMW = low molecular weight chitosan nanoparticles; LAC = chitosan oligosaccharide lactate nanoparticles; SHR = chitosan from shrimp shells nanoparticles; B = bromelain. The backgrounds of the SEM pictures are artefacts introduced from the sample preparation.

Scanning electron microscopy (SEM) was used to evaluate nanoparticles’ morphology. In SEM images (Figure 2), it was possible to confirm the spherical shape with smooth and regular surface of LMW (Figure 2A), LMW-B (Figure 2B), LAC (Figure 2C), LAC-B (Figure 2D), and SHR (Figure 2E) nanoparticles. SHR-B nanoparticles (Figure 2F), however, exhibited nonregular and nonuniform shape and surface. SHR-B image also shows a diffuse network attributed to the non-encapsulated bromelain, as SHR-B nanoparticles showed the lowest encapsulation efficiency.

In SEM images, the results are in agreement with the size distributions recorded by DLS, in which D90 reached ca. 3.5 µm for LAC nanoparticles (Figure 2C, with PdI of 0.541 ± 0.014). The loading of bromelain into SHR nanoparticles also shifted D90 to values above 2 µm (Figure 1) as also seen in Figure 2F. Nevertheless, even the soft drying process, as conducted at room temperature, can lead to nanoparticles aggregation and formation of microparticles [38]. Chitosan has been used for the production of hydrogel nanoparticles through various methods, including ionotropic gelation with TPP, and for several administration routes [13,14,15,16,17,18,39]. These hydrogel-nanoparticulated materials have hydrogel and nanoparticle characteristics simultaneously. Therefore, chitosan nanoparticles present hydrophilicity, flexibility, and high water absorption capacity, as similar to hydrogels [40]. 

**Figure 3:**
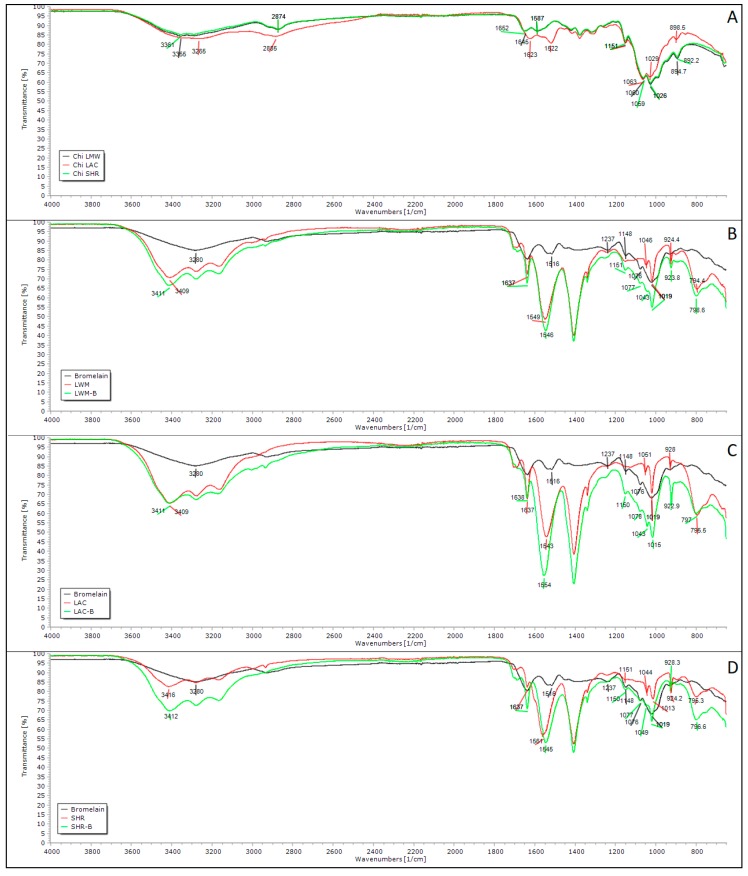
Fourier transform infrared spectra of different types of chitosan (**A**) and nanoparticles prepared with low molecular weight (**B**), oligosaccharide lactate (**C**) and from shrimp shells (**D**) chitosan. LMW = low molecular weight chitosan nanoparticles; LAC = chitosan oligosaccharide lactate nanoparticles; SHR = chitosan from shrimp shells nanoparticles; B = bromelain.

FTIR spectra of different types of chitosan (Figure 3A) show the characteristic polysaccharides peaks in fingerprint region from 1156 to 890 cm^−1^ [41,42], as peaks corresponding to C–H on rings, C–O of alcohols, and C–O–C asymmetric characteristic of glycoside bonds. In chitosan’s spectra it is also possible to observe the peaks around 3300 cm^−1^, corresponding to the hydrogen bonds of O–H groups, which overlaps N–H stretch band [42].

Peaks corresponding to C=O and N–H bonds are present in all samples, however with displacements. For LMW chitosan they appear at 1645 and 1587 cm^−1^, for LAC chitosan at 1622 and 1521 cm^−1^, and for SHR chitosan at 1651 and 1587 cm^−1^, respectively. According to He et al. [43], lower intensity of C=O peaks is associated with higher deacetylation degree. LMW and SHR chitosans presented bands with similar intensity, which is an indication of the same deacetylation degree. LAC chitosan showed a higher intensity band, even being reported to have higher deacetylation grade (> 90%), which may be explained by the presence of C=O in lactate [44]. 

Bromelain exhibits a characteristic enzymatic peptide bond peak at 3280 cm^−1^, and also peaks at 1634 cm^−1^ and 1516 cm^−1^ representing C=O and N–H groups, respectively, and confirming the presence of amino acids. The peak at 1236 cm^−1^ can be attributed to the C–N bond of the aliphatic amine [45,46,47,48]. 

Nanoparticles composed of different chitosans, with and without bromelain, were also analyzed by FTIR. All nanoparticles showed absorption bands around 3400 cm^−1^ equivalent to hydrogen interaction and O–H vibration, with increased intensity when compared to the same peak of different types of chitosans. This result suggests an increase of hydrogen bonds in nanoparticles without bromelain [49]. This same band presents even higher intensity in bromelain nanoparticles. All nanoparticles′ spectra present a peak around 795 cm^−1^, which may be attributed to vibrations related to P–O and P–O–P bonds [50,51].

In the blank nanoparticles, it is possible to observe displacement of C=O and N–H peaks, when compared to the peaks depicted in chitosan spectrum. These displacements indicate interaction between the amino groups of chitosan with the phosphate groups of TPP [50,51]. Comparing this very same peak region, it is possible to note an increased intensity in both peaks in chitosan–bromelain nanoparticles, which can be attributed to bromelain loading into nanoparticles because bromelain also absorbs in the same frequency [45,46]. Chitosan–bromelain nanoparticles also present peaks around 1148 and 1076 cm^−1^ similar to bromelain spectrum, which were not observed in chitosan nanoparticles.

LumiSizer detects light transmitted through the sample in space and time solved by an optoelectronic sensor and determines the instability index the sample. The samples must have a sufficiently high turbidity, which depends on the particle size, shape, the its optical properties. Previous studies showed that initial normalized intensity between 10% and 30% provides maximum reliability, but any transmission value in the range of 5% to 80% is typically acceptable. Freshly prepared nanoparticles showed transmittance profiles higher than 90% during the tested period (data not shown), indicating that backscattering profiles could not be recorded. However, the instability index of all formulations remained below 0.3. A very low instability index values is an indication that particles are physicochemically stable under stress conditions [52,53].

The texture analysis of nanoparticle suspensions was carried out by a back-extrusion test. In this test, maximum force applied is taken as firmness measurement, and greater values indicate a denser sample consistency. The area under the curve up to this point is taken as an indication of the consistency, which refers to “firmness”, “thickness”, or “viscosity” of a liquid or a semisolid. This test also provides an indication of viscosity, which is the result of the sample weight lifted mainly in the disc upper surface on its return, i.e., a measure of resistance to disk flow [54,55].

Cohesion is determined by intermolecular attraction, which maintains together the elements of a body or mass of material. It is related to product internal viscosity and is usually determined by measuring the force required to remove an item from product. In this test, maximum negative force is taken as an indication of the sample’s cohesiveness [54,55].

Nanoparticles made with SHR chitosan presented the lowest viscosity index among all used chitosan types, and this value decreased with the loading of bromelain into the nanoparticles (Table 4). However, for other chitosan types, viscosity index increased with the addition of bromelain into the formulation. The higher cohesiveness was found for LMW chitosan nanoparticles without bromelain, and it decreased with the loading of bromelain. The same behavior was found for other types of chitosan, indicating that bromelain makes the suspension less cohesive.

## 4. Conclusions

Our results confirm that all tested chitosan types (LMW, LAC, and SHR) were suitable for the production of nanoparticles, as demonstrated by dynamic light scattering (DLS) and Fourier transform infrared (FTIR) spectroscopy, resulting in nanoparticles with slightly different physicochemical properties. For all chitosan types, the mean particle size and zeta potential decreased while polydispersity index increased after loading bromelain into the nanoparticles. Bromelain has been successfully loaded within chitosan nanoparticles with desirable high encapsulation efficiency (>84% for proteins, and >79% for enzymatic activity). Bromelain encapsulation in chitosan nanoparticles was also confirmed by FTIR. In general, bromelain addition decreased the cohesiveness of the nanoparticle suspension. Freshly prepared chitosan–bromelain nanoparticles exhibited a low instability index, acceptable for further processing of the suspensions. Based on the obtained results, LMW-chitosan showed a higher capacity for the encapsulation of bromelain, generating moderate polydispersed systems with a spherical shape, with high encapsulation efficiency (89.1% for proteins and 91.9% for enzymatic activity).

## Figures and Tables

**Table 1:** Dynamic light scattering (DLS) conditions used for the measurement of the mean particle size (Z-ave) and polydispersity index (PdI) of nanoparticles.

Parameter	Value
Particles refractive index	1.520
Particles absorption	0.330
Medium viscosity	0.8872 cP
Medium refractive index	1.330
Measurement temperature	25 °C
Cuvette type	Disposable folded capillary cells (DTS1070)
Scattering angle	173°
Laser wavelength	633 nm
Sample preparation prior to analysis	none

**Table 2:** Polydispersity index and zeta potential of chitosan and chitosan–bromelain nanoparticles obtained by DLS, laser Doppler microelectrophoresis, and NTA.

NPs	Polydispersity Index	Zeta Potential (mV)	Concentration (×10^11^ particles/mL)
LMW	0.350 ± 0.051	30.6 ± 2.7	4.7 ± 0.4
LAC	0.541 ± 0.014	28.4 ± 2.4	14.3 ± 0.8
SHR	0.327 ± 0.006	33.2 ± 3.3	9.8 ± 0.2
LMW-B	0.358 ± 0.062	28.9 ± 1.7	6.9 ± 0.3
LAC-B	0.498 ± 0.034	26.3 ± 2.5	19.9 ± 1.3
SHR-B	0.427 ± 0.018	30.0 ± 2.4	1.7 ± 0.8

Results presented as mean ± SD of three measurements. NP = nanoparticles; LMW = low molecular weight chitosan nanoparticles; LAC = chitosan oligosaccharide lactate nanoparticles; SHR = chitosan from shrimp shells nanoparticles; B = bromelain.

**Table 3:** Loading capacity (LC%), encapsulation efficiency (EE%) and enzymatic activity of bromelain loaded in the different types of nanoparticles. The concentration (U/mL) of bromelain in the filtered solution is given as reference.

Sample	Concentration (U/mL) *	LC%	EE (%)	EA (%)
Bromelain Solution	20.4 ± 0.1	-	-	-
LMW-B	1.6 ± 0.5	15.0	89.1	91.9
LAC-B	2.8 ± 0.1	16.4	97.7	86.3
SHR-B	4.1 ± 1.3	14.1	84.1	79.8

LMW = low molecular weight chitosan nanoparticles; LAC = chitosan oligosaccharide lactate nanoparticles; SHR = chitosan from shrimp shells nanoparticles; B = bromelain. * Enzymatic activity in the filtered solution, corresponding to free bromelain.

**Table 4:** Texture analysis of nanoparticles suspensions by back extrusion rig test.

NP	Firmness (g)	Consistency (gs)	Cohesiveness (g)	Viscosity Index (gs)
LMW	980.6 ± 51.9	1629.8 ± 346.6	−909.4 ± 32.0	393.3 ± 386.5
LMW-B	915.9 ± 25.5	1626.4 ± 35.7	−860.8 ± 117.7	412.3 ± 29.3
LAC	935.3 ± 16.5	1695.1 ± 36.3	−906.2 ± 88.4	546.9 ± 108.5
LAC-B	925.6 ± 32.0	1685.1 ± 240.7	−809.1 ± 69.3	637.0 ± 142.7
SHR	873.8 ± 21.0	1444.5 ± 34.4	−851.1 ± 16.5	341.0 ± 41.7
SHR-B	909.4 ± 18.3	1375.4 ± 122.5	−802.6 ± 35.8	266.4 ± 131.2

Results presented as mean ± SD of three measurements. g = grams; s = seconds; LMW = low molecular weight chitosan nanoparticles; LAC = chitosan oligosaccharide lactate nanoparticles; SHR = chitosan from shrimp shells nanoparticles; B = bromelain.
